# Time to maternal death and its predictors among obstetric ICU patients in a resource-limited setting: A 10-year survival analysis

**DOI:** 10.1371/journal.pone.0352904

**Published:** 2026-06-30

**Authors:** Tigist Nega Alemu, Wondimagegn Genaneh Shiferaw, Workneh Elias Orsongo, Solomon Medina Megule, Wondu Feyisa Balcha

**Affiliations:** 1 School of Public Health, Wolaita Sodo University, Sodo City, Ethiopia; 2 Department of Emergency and Critical Care Nursing, Wolaita Sodo University, Sodo City, Ethiopia; 3 Department of Nursing, Bonga University, Bonga, Ethiopia; 4 Department of Midwifery, Bahir Dar University, Bahir Dar, Ethiopia; Kwame Nkrumah University of Science and Technology College of Health Sciences, GHANA

## Abstract

**Background:**

Maternal mortality remains a major concern in resource-limited settings, particularly among critically ill obstetric patients requiring intensive care unit (ICU) admission.

**Objective:**

To assess the time to maternal death and identify its predictors among obstetric patients admitted to the intensive care unit (ICU) in a resource-limited tertiary hospital in southern Ethiopia, over a ten-year period.

**Methods:**

A retrospective cohort study was conducted among 378 obstetric patients admitted to the ICU between 2014 and 2023. Kaplan-Meier analysis estimated survival probability, multivariable Cox proportional hazards regression identified independent predictors of time to maternal death reported as adjusted hazard ratios (AHR) with 95% confidence intervals, and a Fine-Gray competing-risks model was additionally conducted with discharge alive as the competing event.

**Results:**

Of 378 obstetric ICU admissions, 126 resulted in maternal death, with a median time to death of 2.71 days (95% CI: 2.13–3.44); 71.4% of deaths occurred within the first five days, and survival probability declined rapidly before stabilizing after ten days. Rural residency (AHR 1.56, 95% CI 1.03–2.37), shock (AHR 2.27, 95% CI 1.44–3.56), multi-organ failure (AHR 1.75, 95% CI 1.09–2.78), mechanical ventilation (AHR 1.82, 95% CI 1.11–2.98), and impaired consciousness (moderate GCS: AHR 2.94; severe GCS: AHR 5.43, both p < 0.001) were independently associated with higher hazard of death, with findings consistent across the Fine–Gray competing-risks model.

**Conclusion:**

Maternal ICU deaths occurred early, with most fatalities within the first week of admission and survival probability declining sharply in the first ten days. Shock, multi-organ failure, invasive mechanical ventilation, impaired consciousness at admission, and rural residency were independent predictors of shortened time to maternal death. Interventions targeting these conditions must be initiated early and urgently, particularly within the critical first days of ICU admission, to improve survival in resource-limited settings.

## 1. Introduction

According to the World Health Organization (WHO), maternal death is the death of a woman while pregnant or within 42 days of termination of pregnancy, from any cause related to or aggravated by the pregnancy or its management, but not from accidental or incidental causes [1]. Maternal near-miss and severe acute maternal morbidity have recently come to be recognized as indicators of the quality of obstetric treatment [2,3].

Obstetric patients with various medical and surgical emergencies are admitted to an intensive care unit (ICU), which also offers supportive treatment to patients experiencing obstetric problems [4,5]. Approximately 1–9 per 1,000 deliveries require admission to intensive care units (ICUs), with the majority of admission diagnoses being related to obstetric complications [6,7]. Complications such as postpartum hemorrhage, sepsis, eclampsia, and other life-threatening diseases that require sophisticated monitoring and therapeutic measures might receive specialized care in intensive care units [8].

Globally, nearly 800 women die each day from avoidable pregnancy‑related causes, corresponding to a maternal mortality ratio (MMR) of 223 deaths per 100,000 live births [9]. Most maternal mortality occurs in developing countries, with Sub-Saharan Africa (SSA) alone accounting for approximately 70% of these fatalities [10,11]. The financial burden of maternal deaths affects not only the healthcare system but also families and society as a whole [12]. Maternal mortality rates (MMR) in Ethiopia have dramatically decreased, from 871 per 100,000 live births in 2000 (95% CI: 705–1039) to 267 per 100,000 in 2020 (95% CI: 189–427) live births (95 percent CI, 189–427) in 2020 [5,13]. Even though it is trending downward, the maternal mortality ratio is still high in the country [14].To address this, Ethiopia set up the maternal death surveillance and response system (MDSR) with the goal of providing real-time data on the trends and patterns of avoidable maternal deaths [15].

In Ethiopian ICU settings, available evidence remains limited but indicates alarmingly high maternal mortality among obstetric ICU patients: 27% and 29.9% in Addis Ababa, and 17.6% in Mekelle [16–18], which are markedly higher than national averages [5,19], highlighting the critical need for improved care in ICU settings.

Several factors have been consistently associated with increased mortality, including hypertensive disorders, absence of antenatal care (ANC), early ICU discharge, disseminated intravascular coagulation (DIC), HELLP syndrome (Hemolysis, Elevated Liver enzymes, and Low Platelets), vasopressor use, multi-organ failure, preeclampsia, sepsis, advanced maternal age (≥35 years), pre-existing comorbidities, and low Glasgow Coma Scale (GCS) scores [16–18].

Most Ethiopian and sub-Saharan African studies focus on static risk factors using cross-sectional or case-control designs and predominantly include non-ICU patients, despite the fact that the most severe obstetric cases are admitted to intensive care units, providing limited insight into when maternal deaths ensue [11,14,20–24]. Time-to-event (survival) analysis offers a dynamic approach, accounting for censored cases to estimate survival probabilities over time and identify predictors of both the risk and timing of death [25,26]. Survival analyses from Brazil, Nigeria, and South Africa indicate that complications such as shock, low GCS, multi-organ failure, sepsis, postpartum hemorrhage, acute kidney injury, and delayed ICU admission significantly increase mortality and reduce survival times among critically ill obstetric patients. [27–31]. These results highlight how crucial early detection and prompt, crucial interventions are to enhancing maternal outcomes in the critical care unit.

Despite nationwide improvements, maternal mortality among critically ill obstetric patients remains unacceptably high in low-resource settings such as Ethiopia [32]. We recently performed a case–control study of the same obstetric ICU cohort to determine factors associated with maternal mortality [33], which identified several static predictors of mortality, but the case–control design did not examine the temporal aspect of maternal death in the ICU. Survival analysis can be used to estimate time-to-event and to estimate how quickly death occurs and what factors contribute to the hazard of death over time. Such secondary analyses of existing datasets are widely encouraged to generate new scientific insights and maximize the value of previously collected clinical data [34].Therefore, the objective of this study was to use survival analysis to evaluate time to maternal death and its predictors among obstetric patients admitted to the ICU of a tertiary-level Hospital in Southern Ethiopia, over a ten-year period, providing important insights to guide future interventions and policy actions. Understanding when and why maternal death occur in critical care settings can inform resource allocation, optimize timely interventions, and ultimately improve maternal survival.

## 2. Method

### 2.1. Study design and setting

A retrospective cohort study was performed admitted to the intensive care unit of a tertiary-level Hospital in southern Ethiopia. The hospital provides a comprehensive range of outpatient and inpatient services, including emergency, medical, surgical, paediatric, ophthalmic, gynaecological and obstetric care [35]. The hospital encompasses a 10-bed adult Intensive Care Unit (ICU) that receives critically ill patients from all departments, including obstetric cases. It included secondary data of ICU-admitted obstetric patients over ten years.

This study analyzed the same cohort of obstetric ICU patients that was previously used in a case–control study investigating determinants of maternal mortality [33]. However, unlike the previous analysis, which applied a case–control design, the present study employed a survival analysis approach to assess time to maternal death and to identify predictors influencing the hazard of death during ICU admission.

### 2.2. Population and eligibility criteria

The source population comprised all obstetric patients admitted to the intensive care unit (ICU) during the ten-year study period (2014–2023 E.C.). The study participants included women who were pregnant (≥28 weeks of gestation) or within 42 days postpartum and admitted to the ICU due to obstetric or medical complications. Patients with more than 10% missing critical outcome data (admission/discharge status or survival time) were excluded due to inability to determine time-to-event outcomes. Patients referred to other hospitals before outcome determination were also excluded, as no follow-up information was available to ascertain final outcomes or survival time.

### 2.3. Sample size and sampling technique

**Fig 1:**
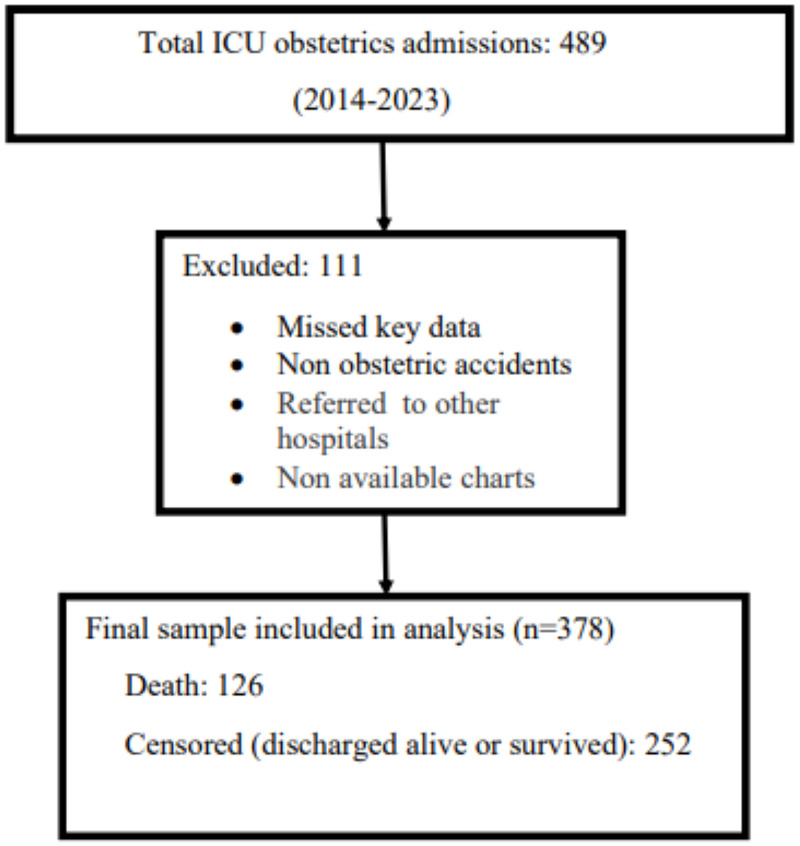
Flow diagram illustrating selection of obstetric ICU patients and data extraction process (2014–2023).

A ten-year total population cohort of obstetric ICU admissions (n = 489) was identified from the hospital registry, and after review for eligibility and data completeness, Finally, 378 patients’ charts (81%) met the established inclusion criteria included in the final analysis (126 deaths and 252 censored cases; exclusions due to critical missing data (n = 111). The sample size was reduced, but it was still adequate for survival analysis using Cox proportional hazards regression. (See Fig 1 for more information).

### 2.4. Variables

#### 2.4.1. Outcome variable.

The outcome variable was time to maternal death, measured in days from ICU admission to the occurrence of death. Patients who were discharged alive were considered censored observations.

#### 2.4.2. Independent variables.

The study considered several factors that could influence maternal outcomes in the ICU. Socio-demographic characteristics included maternal age, residence (urban or rural), and marital status. Clinical factors encompassed gravidity, parity, pre-existing medical conditions, vital signs, and Glasgow Coma Scale (GCS) scores at admission. Interventions assessed during the ICU stay included the use of mechanical ventilation, blood transfusions, dialysis, antenatal care (ANC) follow-up, and other treatments administered in the intensive care unit. Admission-related factors included the primary diagnosis, mode of hospital presentation, and obstetric reason for ICU admission. In addition, complications that developed during the ICU stay, such as shock and multi-organ failure (MOF), were also evaluated as important determinants of maternal death.

### 2.5. Data collection procedure

Data were collected using literature-based checklists [28,36–39] and pretested on a subset of charts in the same hospital and excluded from final analysis. Data were accessed and extracted from ICU log books and patients’ reasonably complete (≥ 90 percent of variables) paper charts between 06/11/2023 and 30/12/2023. The checklists included socio-demographic data, ICU interventions, complications developed during ICU stay, and outcomes. The data were collected from selected participant charts. Two trained health professionals familiar with checklist, confidentiality, standard definitions and confidentiality procedures, performed data extraction with an overall completeness of the data of 96.4%. Quality control was ensured by double-checking a subset of records, and any discrepancies between data collectors were resolved by an assigned supervisor.

### 2.6. Operational definition

Maternal Death (Died): Death of a woman while pregnant or within 42 days of pregnancy termination from causes related to the pregnancy or its managements [1].

Censored (survived) Observations: Patients discharged alive from the ICU were considered censored, while those transferred to another facility were excluded and not censored.

Time to Death: Duration in days from ICU admission to the occurrence of maternal death within the ICU.

Survival Time: The total number of days a patient remained alive in the ICU, ending in either death or discharge.

Shock: Persistent hypotension requiring vasopressor support to maintain a mean arterial pressure (MAP) ≥ 65 mmHg (or equivalent) despite reasonable fluid resuscitation in ICU [40].

Multi-Organ Failures (MOFs): is the development of potentially reversible physiologic derangement involving two or more organ systems not involved in the disorder that resulted in ICU admission, and arising in the wake of a potentially life-threatening physiologic insult [41].

level of consciousness: Assessed on ICU admission using the Glasgow Coma Scale, with scores of 3–8 indicating severely decreased, 9–12 moderately decreased, and ≥13 mildly decreased consciousness [42].

### 2.7. Data management and analysis

Data were first entered into Epi Data version 4.6 and then exported for statistical analysis using SPSS version 25 and Stata MP Version 17.0. Demographic and clinical characteristics of the study population were summarized by descriptive statistics (frequencies, medians, interquartile ranges). Time-to-event data were analyzed with survival analysis techniques; the outcome variable was time to maternal death in days from ICU admission until occurrence of death, and patients who discharged alive were censored at the time of discharge.

We estimated survival probabilities using Kaplan-Meier survival curves, and compared survival distributions between categorical predictors with the log-rank test. A life table analysis was additionally performed to summarize survival experience across predefined time intervals, providing estimates of the number at risk, number of events, censored observations, and interval-specific survival probabilities with corresponding standard errors and confidence intervals. We used a Cox proportional hazards regression model to identify independent predictors of maternal mortality: bi-variable Cox regression for each potential predictor, all variables with a p-value < 0.25 in the bi-variable analysis and clinically important variables included in the multivariable Cox regression model (reported as adjusted hazard ratios [AHR] with 95% confidence intervals [CI]). The proportional hazards assumption was assessed using log-minus-log survival plots and Schoenfeld residuals. The global Schoenfeld test indicated no violation of the proportional hazards assumption (p = 0.781), and it was found to be satisfied. Among the included participants, 96.4% had complete data. To account for the competing risk of discharge alive, a Fine-Gray sub distribution hazards model was additionally conducted using the same covariates, with results reported as sub distribution hazard ratios (SHR) with 95% confidence intervals. The remaining 3.6% of missing values were observed in selected covariates such as age, marital status, and other socio-demographic and clinical variables included in the regression analysis. These missing values were handled using single imputation, applying median imputation for continuous variables and mode imputation for categorical variables. Model adequacy was assessed by examining Cox-Snell residuals to determine whether the Cox proportional hazards model appropriately fit the data. A p-value < 0.05 was considered statistically significant in the final model.

### 2.8. Ethical considerations

The study was granted ethical approval by the University Ethical Review Committee, with protocol number CHSM/ERC/01/16. Informed consent was waived by the ERC because the study used retrospectively collected, anonymized chart data.

## 3. Results

### 3.1. Demographic distribution of obstetric ICU admissions of the study

Out of 489 initial obstetrics ICU admissions over 10 years, the final cohort of 378 patients to be analyzed met the eligibility criteria and had complete medical records. The age of obstetric patients who died ranged from 18 to 45 years, while that of the survivors (censored cases) ranged from 18 to 48 years. The mean age for both cohorts was determined to be 29 years; however, the interquartile range (IQR) was more extensive for those who died (8.25 years) compared to the censored participants (5.75 years). A significant proportion of patients consisted of 103 (81.7%) of those who died and 218 (86.5%) of those who were censored, with the majority falling within the 18–34-year age range.

Most of the deceased patients resided in rural locations (88, 69.9%), while more of the censored participants were from urban areas (144, 57.2%). For both groups, about two-thirds of the participants were married, and around 3% were widowed. (See Table 1 for more information)

**Table 1:** Socio-demographic profile of obstetric ICU patients by outcome status (death vs. censored), Tertiary level Hospital, Southern Ethiopia, 2014–2023.

Variable	Total (n = 378)	Died (n = 126)	Censored) (n = 252)
Age group (years)			
18–24	124 (32.8%)	42 (33.3%)	82 (32.5%)
25–34	197 (52.1%)	61 (48.4%)	136 (54.0%)
≥ 35	57 (15.1%)	23 (18.3%)	34 (13.5%)
Median age (IQR)	29 (6.5)	29 (8.25)	29 (5.75)
Residence			
Urban	146 (38.6%)	38 (30.1%)	108 (42.8%)
Rural	232 (61.4%)	88 (69.9%)	144 (57.2%)
Marital status			
Married	252 (66.7%)	84 (66.7%)	168 (66.7%)
Single	110 (29.1%)	38 (30.2%)	72 (28.6%)
Widowed	11 (2.9%)	4 (3.2%)	7 (2.8%)
Other	5 (1.3%)	0 (0.0%)	5 (2.0%)

Data are presented as n (%) or median (IQR). Subcategories are indented for clarity.

### 3.2. ICU admission characteristics and primary diagnoses of obstetric patients

The timing of ICU admission, length of stay, and types of diagnoses differed between the two groups (Died vs Censored).

Concerning admission timing, more maternal deaths happened after childbirth (postpartum); 65.9% of deaths occurred during this period, compared to 50.8% of survivors (censored). Conversely, 44.5% of censored patients were admitted during the pregnant period (antenatal), while only 29.4% of those who died were admitted during pregnancy.

The median time to death was 2.7 days (95% CI: 2.1–3.4), and 71.4% of deaths occurred within the first 5 days, but only 52.7% of survivors (censored) stayed fewer than five days. This shows that most deaths happened soon after ICU admission.

About admission diagnoses, obstetric hemorrhage was more frequent in patients who died (41.2%) than in those who survived (28.6%). Likewise, shock and multi-organ failure were reported more often in maternal deaths (46.0% and 20.6%, respectively) than in censored patients (15.1% and 4.8%, respectively).

On the other hand, hypertensive disorders of pregnancy were about the same in both groups, while heart problems were more frequent among survivors (34.1%) than in maternal deaths (23.0%). Severe anemia was common in both groups, affecting 54.0% of maternal deaths and 51.2% of censored patients. (See Table 2 for more information).

**Table 2:** Clinical presentation and primary ICU diagnoses of obstetric patients by outcome at a Tertiary-level Hospital in Southern Ethiopia, 2014-2023.

Variable	Died (n = 126)	Survived(Censored) (n = 252)	Total (n = 378)
Presentation at Admission			
Pregnant	37 (29.4%)	112 (44.5%)	149 (39.4%)
Postpartum	83 (65.9%)	128 (50.8%)	211 (55.8%)
Post-abortion	6 (4.7%)	12 (4.7%)	18 (4.8%)
Length of ICU Stay			
< 5 days	90 (71.4%)	133 (52.7%)	223 (59.0%)
≥ 5 days	36 (28.6%)	119 (47.3%)	155 (41.0%)
Obstetric Admission Diagnoses			
Severe anemia	68 (54.0%)	129 (51.2%)	197 (52.1%)
Obstetric hemorrhage	52 (41.2%)	72 (28.6%)	124 (32.8%)
Hypertensive disorders	42 (33.3%)	82 (32.5%)	124 (32.8%)
Non-Obstetric Admission Reasons			
Respiratory disorders	33 (26.1%)	55 (21.8%)	88 (23.3%)
Cardiac disorders	29 (23.0%)	85 (34.1%)	114 (30.2%)
Renal disorders	23 (18.2%)	42 (16.7%)	65(17.19)

### 3.3. Management and clinical characteristics of ICU-admitted obstetric mothers: Comparison between died and censored groups

In our study of obstetric patients admitted to the ICU, most received antibiotics: 94.4% of those who died and 98.4% of those who survived (censored). Blood transfusions were also common, with almost three-quarters of those who died (73.8%) and about two-thirds of survivors (63%). Obstetric patients who died were much more likely to receive intensive life-saving interventions: CPR was performed on 14.2%, dialysis on 10.3%, and Invasive mechanical ventilation on 80% compared with lower rates for the survivors (censored), (11.1% CPR, 3.1% dialysis, and 43.2% mechanical ventilation).

Many of the patients who died showed signs of serious illness: about one third was tachycardic (heart rate, above 100 beats per minute) and hypertensive (blood pressure above 130/80 mmHg) was common. Nearly 40% were unconscious on admission, reflected by low scores on the Glasgow Coma Scale. (See Table 3 for more information).

**Table 3:** Management and clinical characteristics of obstetric ICU patients by survival outcome at a Tertiary-level care ICU, 2014-2023.

Variable	Categories	Died n (%)	Censored n (%)	Total n(378)
Antibiotic given	Yes	119 (94.4)	248 (98.4)	367 (97.1)
	No	7 (5.6)	4 (1.6)	11 (2.9)
Blood Transfused	Yes	93 (73.8)	159 (63.1)	252 (66.7)
	No	33 (26.2)	93 (36.9)	126 (33.3)
Anticoagulant given	Yes	25 (19.8)	54 (21.4)	79 (20.9)
	No	101 (80.2)	198 (78.6)	299 (79.1)
Catecholamine given	Yes	17 (13.5)	25 (9.9)	42 (11.1)
	No	109 (86.5)	227 (90.1)	336 (88.9)
Inotrope given	Yes	1 (0.8)	2 (0.8)	3 (0.8)
	No	125 (99.2)	250 (99.2)	375 (99.2)
CPR Performed	Yes	18 (14.3)	28 (11.1)	46 (12.2)
	No	108 (85.7)	224 (88.9)	332 (87.8)
Dialysis Performed	Yes	13 (10.3)	8 (3.2)	21 (5.6)
	No	113 (89.7)	244 (96.8)	357 (94.4)
Oxygen via Nasal Cannula/FM	Yes	89 (70.6)	191 (75.8)	280 (74.1)
	No	37 (29.4)	61 (24.2)	98 (25.9)
Invasive Mechanical Ventilation	Yes	101 (80.2)	109 (43.3)	210 (55.6)
	No	25 (19.8)	143 (56.7)	168 (44.4)
Mannitol Given	Yes	4 (3.2)	6 (2.4)	10 (2.6)
	No	122 (96.8)	246 (97.6)	368 (97.4)
Diuretic given	Yes	31 (24.6)	54 (21.4)	85 (22.5)
	No	95 (75.4)	198 (78.6)	293 (77.5)
Magnesium Sulfate given	Yes	44 (34.9)	70 (27.8)	114 (30.2)
	No	82 (65.1)	182 (72.2)	264 (69.8)
Hysterectomy conducted	Yes	7 (5.6)	16 (6.3)	23 (6.1)
	No	119 (94.4)	236 (93.7)	355 (93.9)
Cesarean Section performed	Yes	4 (3.2)	7 (2.8)	11 (2.9)
	No	122 (96.8)	245 (97.2)	367 (97.1)
Baseline Pulse Rate	Bradycardic	22 (17.4)	34 (13.5)	56 (14.8)
	Normal	64 (50.8)	147 (58.3)	211 (55.8)
	Tachycardic	40 (31.7)	71 (28.2)	111 (29.4)
Baseline Blood Pressure	Hypotensive	23 (18.1)	47 (18.7)	70 (18.5)
	Normal	55 (43.3)	112 (44.5)	167 (44.2)
	Hypertensive	48 (37.6)	93 (36.9)	141 (37.3)
Baseline Oxygen Saturation	Normal	60 (47.2)	129 (51.4)	189 (50.0)
	Hypoxic	66 (52.8)	123 (48.6)	189 (50.0)
Baseline GCS	Severe	90 (70.9)	54 (21.5)	144 (38.1)
	Moderate	12 (9.5)	98 (39.0)	110 (29.1)
	Mild/Normal	24 (18.9)	100 (39.5)	124 (32.8)

Shock was the most frequent complication, affecting 65% of patients who died, mostly due to blood loss (hypotension), whereas fewer (33%) survivors (censored) experienced shock. Around one fourth of each group faced infections acquired during their ICU stay (Healthcare associated- infection), but multi-organ failure was much more frequent among non-survivor mothers (over 20%) than those who survived (5%). Respiratory dysfunction was the common disorder in ICU- admitted obstetric cases; those who died 36 (28.6%), and survivors had 59 (23.4%). (See Table 4 for more information).

**Table 4:** ICU complications and organ dysfunction in obstetric patients, comparison by survival outcome in a Tertiary-level Hospital, Southern Ethiopia, and 2014–2023.

Variable	Category	Died n (%)	Censored n (%)	Total n(378)
Shock	Yes	82 (65.1%)	67 (26.6%)	149 (39.4%)
	No	44 (34.9%)	185 (73.4%)	229 (60.6%)
DIC(Disseminated Intravascular Coagulation)	Yes	22 (17.5%)	114 (45.2%)	136 (36.0%)
	No	104 (82.5%)	138 (54.8%)	242 (64.0%)
MOF(Multiple Organ Failure)	Yes	27 (21.4%)	99 (39.3%)	126 (33.3%)
	No	99 (78.6%)	153 (60.7%)	252 (66.7%)
Healthcare-Associated Infections (HAIs)	Yes	32 (25.4%)	110 (43.7%)	142 (37.6%)
	No	94 (74.6%)	142 (56.3%)	236 (62.4%)
Cardiac Dysfunction	Yes	32 (25.4%)	90 (35.7%)	122 (32.3%)
	No	94 (74.6%)	162 (64.3%)	256 (67.7%)
Respiratory Dysfunction	Yes	36 (28.6%)	59 (23.4%)	95 (25.1%)
	No	90 (71.4%)	193 (76.6%)	283 (74.9%)
Renal Dysfunction	Yes	26 (20.6%)	44 (17.5%)	70 (18.5%)
	No	100 (79.4%)	208 (82.5%)	308 (81.5%)
Endocrine Dysfunction	Yes	6 (4.8%)	21 (8.3%)	27 (7.1%)
	No	120 (95.2%)	231 (91.7%)	351 (92.9%)
Neurologic Dysfunction	Yes	20 (15.9%)	27 (10.7%)	47 (12.4%)
	No	106 (84.1%)	225 (89.3%)	331 (87.6%)
Infections (other than HAIs)	Yes	2 (1.6%)	22 (8.7%)	24 (6.3%)
	No	124 (98.4%)	230 (91.3%)	354 (93.7%)

### 3.4. Survival outcomes and time to maternal death of obstetric patients at the tertiary-level hospital of South Ethiopia

Among the 378 obstetric ICU admissions, 126 patients (33.3%) died, while 252 (66.7%) were discharged alive and considered censored cases in the survival analysis.

The mean survival time was estimated at 24.6 days (95% CI: 17.5–31.7). The median survival time estimate of 21 days (95% CI: 4.4–37.6). The median is lower than the mean because of right censoring: patients who were discharged alive contributed survival time only up to the date of discharge, but did not experience the event (death).

### 3.5. Time-to-event of ICU-admitted obstetric patients at a tertiary-level hospital, South Ethiopia

**Fig 2:**
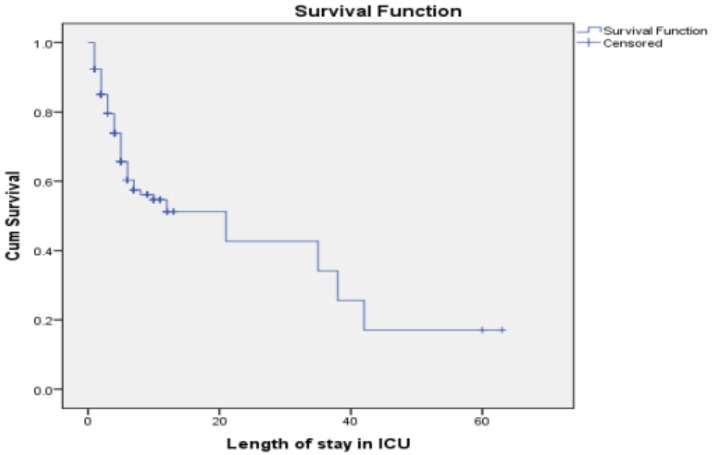
Kaplan-Meier survival curve for ICU length of stay of participants at Tertiary level Hospital of Southern Ethiopia, 2014-2023.

**Fig 3:**
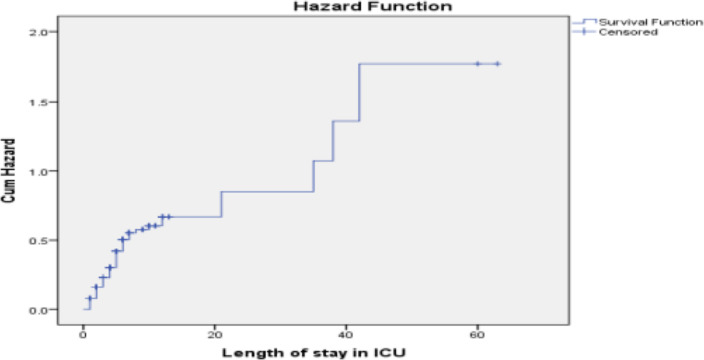
Kaplan-Meier hazard curve for ICU length of stay of participants at Tertiary- level Hospitals in southern Ethiopia, 2014-2023.

The Kaplan-Meier survival curve showed that a significant proportion of patients experience the event of interest (died) early during their ICU stay. Specifically, the survival probability drops sharply within the first 10 days, with approximately 60% of patients experiencing the event during this early period. Beyond 30 days, the survival probability declines more gradually, stabilizing at around 20% by day 60. (See Fig 2, Fig 3 and Table 5 for more information).

**Table 5:** Life table of time-to-event (survival) analysis among ICU-admitted obstetric patients in a Tertiary-level Hospital, Southern Ethiopia.

ICU stay (days)	At risk	Deaths	Discharged	Survival	Std. error	95% CI
**0–5**	378	91	134	0.7074	0.0258	0.6534 - 0.7546
**5–10**	153	29	87	0.5200	0.0353	0.4486- 0.5867
**10–15**	37	2	29	0.4738	0.0448	0.3839- 0.5584
**15–20**	–	–	–	–	–	–
**20–25**	6	1	0	0.3949	0.0812	0.2384- 0.5476
**25–35**	–	–	–	–	–	–
**35–40**	5	2	0	0.2369	0.0993	0.0783- 0.4430
**40–45**	3	1	0	0.1579	0.0924	0.0322- 0.3710
**45–60**	–	–	–	–	–	–
**60–65**	2	0	2	0.1579	0.0924	0.0322- 0.3710

The life table confirms that the majority of deaths occurred during the early ICU stay, particularly within the first 5–10 days, with a progressively decreasing survival probability over time.

### 3.6. Test for equality of survival function of the event of interest of the study participants

A Log-rank test was conducted to see the difference in survival time between different predictors. Accordingly, variables including baseline GCS score, shock, and multi-organ failure had significant survival differences.

The log-rank test (Mantel-Cox) indicated a statistically significant difference in survival distributions across the three GCS categories (χ² = 78.641, df = 2, p < 0.001). This suggests that the initial GCS score on ICU admission is strongly associated with survival outcomes, with patients in the lower GCS categories experiencing significantly poorer survival compared to those with higher scores.

There was also a significant survival difference between patients with and without shock (χ² = 32.659, df = 1, p < 0.001). Patients who developed shock after ICU admission had significantly lower survival probabilities compared to those without shock, indicating that shock is a strong predictor of mortality in critically ill patients.

**Fig 4:**
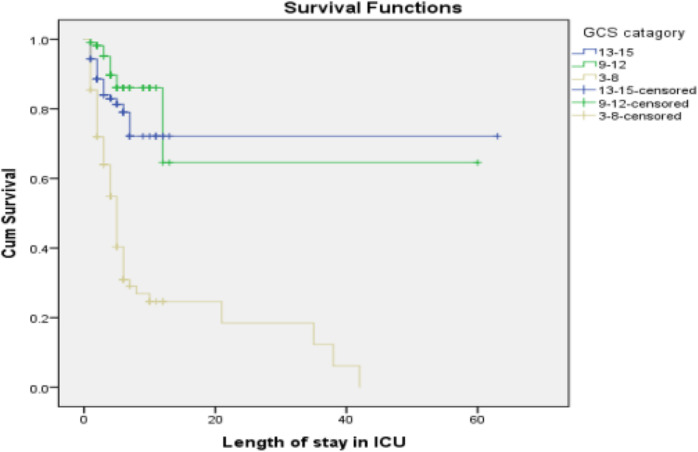
Kaplan-Meier survival estimates by Glasgow Coma Scale (GCS) categories among ICU patients at Tertiary-level Hospital of southern Ethiopia, 2014-2023.

**Fig 5:**
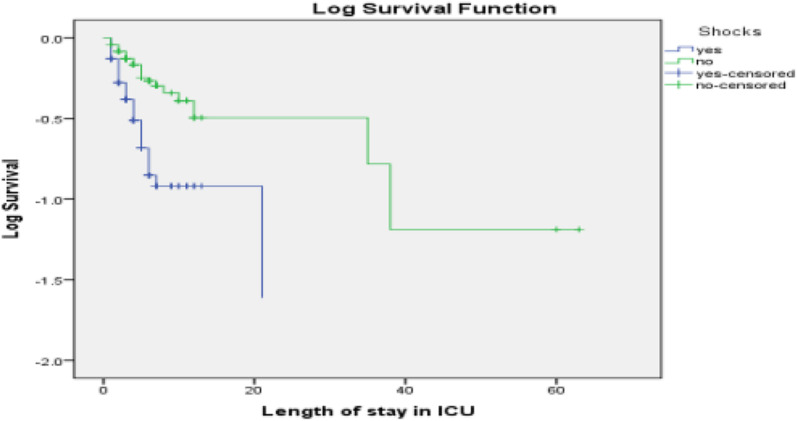
Kaplan-Meier survival curve comparing ICU patients with and without shock at Tertiary-level Hospital, 2014-2023.

**Fig 6:**
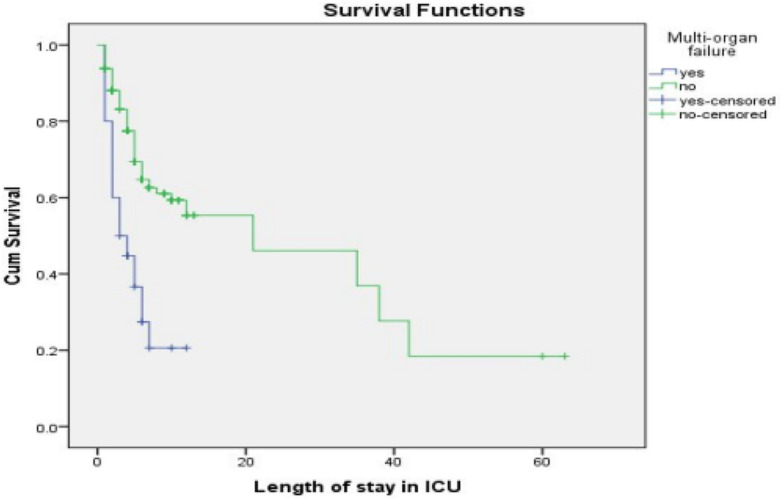
Kaplan-Meier survival curve comparing ICU patients with and without multi-organ failure at a Tertiary-level Hospital of Southern Ethiopia, 2014-2023.

Furthermore, a statistically significant difference in survival distributions based on the development of multi-organ failure (χ² = 29.339, df = 1, p < 0.001). Patients who developed multi-organ failure had significantly lower survival compared to those without, highlighting MOF as a critical predictor of mortality during ICU stay. (See Figs 4–6 for more information).

### 3.7. Independent predictors of maternal mortality among obstetric ICU patients: Multivariable Cox regression analysis and competing-risks regression analysis

Multivariable Cox proportional hazards regression was conducted to determine independent predictors of maternal death among obstetric ICU patients. Variables that had a p-value less than 0.25 in bivariate analysis were included in the model.

In the bivariate Cox regression analysis, several factors were significantly associated with maternal mortality, including age, residence, presentation at admission, obstetric hemorrhage, HELLP syndrome, amniotic fluid embolism, gestational diabetes mellitus, cardiac disorders, respiratory disorders, use of antibiotics, use of catecholamine, administration of anticoagulants, blood product transfusions, dialysis, invasive mechanical ventilation, decreased level of consciousness (GCS), healthcare-associated infections, shock, and multi-organ failure.

After adjusting for potential confounders, several variables were found to be significantly associated with time to maternal death. Rural residency was associated with a significantly higher risk of maternal death compared to urban residency (AHR = 1.563, 95% CI: 1.026–2.371, p = 0.036). Mechanical ventilation increased the hazard of maternal death by 81.9% (AHR = 1.819, 95% CI: 1.109–2.983, p = 0.018), while having multiple organ failure (MOF) after ICU admission was associated with a 74.6% higher hazard of death (AHR = 1.746, 95% CI: 1.094–2.786, p = 0.019).

One of predictors for decreased survival was low levels of consciousness upon admission. Compared to patients with mild GCS scores (13–5), those with moderate GCS scores (9–12) had nearly a threefold higher hazard of death (AHR = 2.942, 95% CI: 1.793–4.833, p < 0.001), and patients with severe GCS scores (<9) had nearly a five folds higher hazard of death (AHR = 5.431, 95% CI: 2.903–10.124, p < 0.001). Additionally, the presence of shock after ICU admission doubled the hazard of maternal death (AHR = 2.267, 95% CI: 1.421–3.559, p < 0.001).

To account for the competing risk of discharge alive, a multivariable Fine-Gray sub-distribution hazards model was conducted using the same covariates, with discharge alive specified as the competing event. Of 378 patients, 126 experienced ICU death and 252 were discharged alive, no observations were censored in the competing-risks model.

Four predictors retained independent significance in the Fine–Gray model. Rural residency (SHR 1.55, 95% CI 1.03–2.33, p = 0.035), severe impairment of consciousness at admission (GCS < 9; SHR 2.82, 95% CI 1.66–4.80, p < 0.001), shock (SHR 2.99, 95% CI 1.97–4.55, p < 0.001), and invasive mechanical ventilation (SHR 2.27, 95% CI 1.39–3.73, p = 0.001) were each independently associated with a higher sub-distribution hazard of ICU mortality. Moderate GCS impairment (9–12) showed a borderline non-significant trend (SHR 0.50, 95% CI 0.25–1.01, p = 0.053). Multi-organ failure did not retain significance (SHR 1.55, 95% CI 0.95–2.54, p = 0.079). (See Table 6 for more information).

**Table 6:** Bivariate, Multivariable Cox proportional hazards regression and fine-gray competing-risks regression analyses for predictors of time to maternal death (n = 378) of the Tertiary-level Hospital in southern Ethiopia, 2014-2023.

Predictor Variable	Category	Died 126 (%)	Censored 252 (%)	CHR (95% CI)	p-value	AHR (95% CI)	p-value	SHR (95% CI)	p-value
**Age**	**18–34**	103 (81.7%)	170 (67.5%)	1	–	1	–	1	–
	**≥35**	23 (18.3%)	82 (32.5%)	0.72 (0.46–1.14)	0.166	1.21 (0.79–2.03)	0.476	0.97 (0.96–1.03)	0.810
**Residency**	**Urban**	38 (30.1%)	108 (42.8%)	1	–	1	–	1	–
	**Rural**	88 (69.9%)	144 (57.2%)	1.28 (1.06–1.55)	0.036	1.56 (1.03–2.37)	0.039*	1.55 (1.03–2.33)	0.035*
**Presentation at admission**	**Pregnant**	37 (29.4%)	112 (44.5%)	1	–	1	–	1	–
	**Postpartum**	83 (65.9%)	128 (50.8%)	1.21 (0.88–1.67)	0.248	0.94 (0.66–1.34)	0.740	1.08 (0.78–1.50)	0.650
	**Post-abortion**	6 (4.7%)	12 (4.7%)	0.77 (0.54–1.09)	0.145	0.96 (0.66–1.40)	0.832	1.08 (0.78–1.50)	0.741
**Obstetric Hemorrhage**	**Yes**	52 (41.2%)	72 (28.6%)	1.30 (0.91–1.86)	0.150	0.86 (0.54–1.38)	0.545	1.39 (0.85–2.27)	0.190
	**No**	74 (58.8%)	180 (71.4%)	1	–	1	–	1	–
**HELLP syndrome**	**Yes**	18 (14.3%)	46 (18.3%)	1.89 (0.99–3.61)	0.054	1.40 (0.65–3.03)	0.391	0.77 (0.39–1.52)	0.453
	**No**	108 (85.7%)	206 (81.7%)	1	–	1	–	1	–
**Cardiac Disorders**	**Yes**	32 (25.4%)	90 (35.7%)	0.77 (0.51–1.177)	0.230	1.15 (0.71–1.84)	0.571	0.86 (0.58–1.27)	0.440
	**No**	94 (74.6%)	162 (64.3%)	1	–	1	–	1	–
**Respiratory Disorders**	**Yes**	36 (28.6%)	59 (23.4%)	1.49 (0.94–2.35)	0.086	1.51 (0.96–2.39)	0.077	0.82 (0.50–1.35)	0.444
	**No**	90 (71.4%)	193 (76.6%)	1	–	1	–	1	–
**Antibiotics**	**Yes**	119 (94.4%)	248 (98.4%)	0.39 (0.18–0.84)	0.016	0.62 (0.25–1.52)	0.295	2.03 (0.92–4.49)	0.080
	**No**	7 (5.6%)	4 (1.6%)	1	–	1	–	1	–
**Catecholamines**	**Yes**	17 (13.5%)	25 (9.9%)	0.92 (0.51–1.64)	0.772	0.93 (0.52–1.66)	0.798	0.98 (0.62–1.55)	0.924
	**No**	109 (86.5%)	227 (90.1%)	1	–	1	–	1	–
**Anticoagulants**	**Yes**	25 (19.8%)	54 (21.4%)	0.65 (0.38–1.12	0.124	0.67 (0.38–1.15)	0.140	1.46 (0.96–2.22)	0.080
	**No**	101 (80.2%)	198 (78.6%)	1	–	1	–	1	–
**Blood Product Transfusions**	**Yes**	93 (73.8%)	159 (63.1%)	1.46 (0.99–2.18)	0.062	1.01 (0.61–1.67)	0.975	0.84 (0.56–1.28)	0.423
	**No**	33 (26.2%)	93 (36.9%)	1	–	1	–	1	–
**Dialysis**	**Yes**	13 (10.3%)	8 (3.2%)	1.85 (1.03–3.33)	0.039	1.75 (0.80–3.84)	0.159	0.92 (0.45–1.90)	0.827
	**No**	113 (89.7%)	244 (96.8%)	1	–	1	–	1	–
**Invasive Mechanical Ventilation**	**Yes**	101 (80.2%)	109 (43.3%)	1.79 (1.43–2.23)	<0.001	1.82(1.11–2.98)	0.018*	2.27 (1.39–3.73)	0.001*
	**No**	25 (19.8%)	143 (56.7%)	1	–	1	–	1	–
**GCS**	**Mild/Normal (13–15)**	24 (18.9%)	100 (39.5%)	1	–	1	–	1	–
	**Moderate (9–12)**	12 (9.5%)	98 (39.0%)	3.56 (2.26–5.59)	<0.001	2.94 (1.79–4.83)	<0.001*	0.50 (0.25–1.01)	0.053
	**Severe (3–8)**	90 (70.9%)	54 (21.5%)	6.67 (3.64–12.20)	<0.001	5.43 (2.90–10.12)	<0.001*	2.82 (1.66–4.80)	<0.001*
**HAIs**	**Yes**	32 (25.4%)	110 (43.7%)	0.82 (0.63–1.06)	0.139	0.82 (0.63–1.06)	0.123	1.08 (0.70–1.67)	0.731
	**No**	94 (74.6%)	142 (56.3%)	1	–	1	–	1	–
**Shock**	**Yes**	82 (65.1%)	67 (26.6%)	1.64 (1.37–1.97)	<0.001	2.27 (1.42–3.56)	0.001*	2.99 (1.97–4.55)	<0.001*
	**No**	44 (34.9%)	185 (73.4%)	1	–	1	–	1	–
**MOF**	**Yes**	27 (21.4%)	99 (39.3%)	1.72(1.39–2.13)	0.019	1.75 (1.09–2.79)	0.019*	1.55 (0.95–2.54)	0.079
	**No**	99 (78.6%)	153 (60.7%)	1	–	1	–	1	–

**Note:** CHR = Crude Hazard Ratio; AHR = Adjusted Hazard Ratio; SHR = Sub-distribution Hazard Ratio; CI = Confidence Interval; GCS = Glasgow Coma Scale; HAIs = Hospital-Acquired Infections; MOF = Multi-Organ Failure. AHR and SHR are adjusted for all variables listed in the table. * Statistically significant at p < 0.05 in the multivariable model.

## 4. Discussion

In our study, the median survival time was 21 days (95% CI: 4.4–37.6), and the Kaplan-Meier survival curve showed a steep decline during the early days of ICU admission, indicating that many maternal deaths occurred shortly after entering intensive care. Although a specific peak mortality period was not defined, the shape of the survival curve suggests a high risk of early death among critically ill obstetric patients. Similar patterns have been observed in other studies from sub-Saharan Africa, where early ICU mortality is often linked to delayed presentation and the limited capacity of critical care units. A study in Nigeria found that a substantial number of maternal deaths occurred soon after ICU admission, primarily among patients with shock and multi-organ failure [28]. Similarly, a study conducted in South Africa reported that septic obstetric patients admitted to the ICU had high early mortality, especially within the first three days [29]. An Ethiopian study also showed that most maternal ICU deaths happened within the first week of admission, further supporting the importance of timely care [18]. These findings highlight the need for early risk stratification, prompt transfer to the ICU, and aggressive management of critically ill obstetric patients during the initial phase of care in resource-limited settings.

The study also identified key independent predictors of maternal mortality among obstetric patients admitted to the ICU. The multivariable Cox regression analysis revealed that rural residency, invasive mechanical ventilation, developed multiple organ failure (MOF), poor Glasgow Coma Scale (GCS) scores upon admission, and having post admission shock were significant predictors of increased hazard of maternal death.

The finding that rural residency was associated with lower survival aligns with previous Ethiopian studies, which attribute poorer outcomes to limited access to timely and quality obstetric care in rural areas [43]. In rural settings, delays in recognizing complications and reaching healthcare facilities significantly contribute to increased maternal mortality [44]. This disparity underscores the ongoing need for improved referral systems and enhanced critical care accessibility for rural populations.

The increased hazard of death associated with invasive mechanical ventilation reflects the severity of illness among those requiring this intervention. Mechanical ventilation is commonly used as a marker of critical respiratory failure or multisystem involvement in ICU patients, and it has been consistently reported as a predictor of mortality in obstetric ICU cohorts worldwide [45,46]. These findings highlight the importance of early identification and management of respiratory compromise to improve outcomes.

In the study, low GCS scores (< 9) upon admission also found to be predictor of mortality, underscoring the prognostic value of neurological status in critically ill obstetric patients. A low GCS typically reflects underlying cerebral hypoxia, severe metabolic derangements, or intracranial pathology, all of which compromise systemic organ perfusion and increase vulnerability to death [47]. Similar findings have been reported in Ethiopian tertiary hospitals and other resource-limited settings where delayed management of neurological complications leads to poor outcomes [48–51]. These findings emphasize the need for early neurological assessment and continuous monitoring. In low-resource settings, constraints such as limited neuroimaging, trained personnel, and delayed referrals impede timely intervention, contributing to higher mortality [8,52]. Strengthening training, resources, and protocol-driven care could markedly improve outcomes for critically ill obstetric patients.

Shock in ICU, predominantly hemorrhagic in origin, more than doubled the hazard of death in this cohort. This is consistent with other obstetric ICU studies, which identify shock as a critical determinant of maternal mortality due to its rapid progression and high fatality if not promptly managed [19,53]. Effective management of shock, including blood transfusion and hemodynamic support, remains a cornerstone of improving survival [54]. In resource-limited ICUs, delayed recognition, limited availability of blood products, and restricted access to advanced monitoring often exacerbate outcomes [55]. Similarly, developed multi-organ failure (MOF) in ICU significantly increases the risk of mortality, as shown in both the Ethiopian and the global ICU studies, reflecting the cumulative effects of serious conditions such as sepsis, hemorrhage, and shock [56–59]. In resource-limited settings, delayed recognition and inadequate availability of advanced supportive care, such as vasopressors, renal replacement therapy, and invasive monitoring, further worsen outcomes [8,60]. Shock causes tissue hypo-perfusion and cellular hypoxia, leading to metabolic acidosis and organ dysfunction [61]. Persistent hypoxia and systemic inflammation trigger multi-organ failure, impairing vital organ function. Without timely intervention, these processes culminate in irreversible organ failure and death [62,63]. Both findings highlight the common underlying problem of insufficient critical care capacity and highlight the need for protocol-based management, early detection of fragility, and investment in ICU infrastructure and staff training to improve maternal outcomes in resource-poor settings.

Our findings are consistent with our previous study conducted using the same cohort [33], which identified developed Shock, Multi-organ failure, decreased level of consciousness (GCS) and invasive mechanical ventilation as key predictors of maternal mortality. While the earlier study examined static associations, the present survival analysis incorporates the temporal dimension by evaluating time to maternal death. Assessing the timing of death provides additional insight into how rapidly these clinical conditions influence mortality risk [64,65]. The consistency of predictors across both analytical approaches strengthens the robustness of these findings among obstetric ICU patients [34].

The application of the Fine–Gray competing-risks model largely corroborated the Cox model findings, with rural residency, severe impairment of consciousness, shock, and mechanical ventilation retaining significance across both models, strengthening confidence in these associations. However, Multi-organ failure remained a significant predictor in the cause-specific Cox model but lost statistical significance in the Fine–Gray competing-risks model (SHR = 1.55, 95% CI: 0.95–2.54; p = 0.079). This difference likely reflects the influence of the competing event of discharge alive on the cumulative incidence of mortality. While the Cox model estimates the instantaneous risk of death among patients remaining at risk in the ICU, the Fine–Gray model incorporates the probability of discharge alive, which may attenuate the observed effect of multi-organ failure on mortality. These findings suggest that competing-risk methods provide additional insight into ICU outcomes in settings where discharge alive is a common and clinically meaningful event [66].

Overall, these findings illustrate that despite contextual differences, the determinants of maternal ICU mortality are largely consistent worldwide, while the magnitude of risk is intensified in low-resource environments. Addressing these challenges requires system-level interventions, including expanding ICU capacity, implementing protocol-driven management for obstetric emergencies, ensuring blood product availability, and enhancing early detection of critical illness. Strengthening referral linkages and investing in ICU staff training, particularly in rural and secondary facilities, are essential steps toward reducing preventable maternal deaths.

## 5. Strengths and limitations

This study is the first cohort analysis of obstetric ICU patients in the study area, providing important insights into maternal survival and its predictors using a 10-year dataset. Survival analysis strengthened the methodology by appropriately handling time-to-event data. The inclusion of a Fine -Gray competing-risks model alongside the Cox proportional hazards model is a key strength, as it accounts for discharge alive as a competing event and provides more robust estimates of mortality risk.

As a retrospective study, the findings may be affected by information bias due to incomplete or inaccurate medical records and limited availability of some clinical variables. Missing data were addressed using single imputation, which may underestimate variability and introduce bias if data were not missing completely at random. In addition, exclusion of patients with incomplete outcome data may have contributed to selection bias.

The single-center design limits external validity and generalizability to other settings. Despite these limitations, the study addresses a critical gap in obstetric critical care in low-resource settings and provides evidence to inform future research and practice.

## 6. Conclusion and recommendations

The study showed a high early maternal mortality among obstetric patients admitted to the ICU, with over two-thirds of deaths occurring in the first five days of admission and a short median time to death. Independent clinical factors associated with an increased hazard of maternal death included complications such as shock and organ failure, the need for invasive mechanical ventilation, rural residency, and low consciousness at admission, highlighting the importance of early recognition, rapid resuscitation, and proactive critical care for high-risk obstetric patients.

Standardized protocols for prompt identification and aggressive management of life-threatening complications such as neurological impairment, hemodynamic instability, and respiratory failure; optimization of mechanical ventilation; strengthening of triage systems; and ensuring equal access to intensive care, particularly for patients from rural areas, are the key priorities to improve outcomes. Given the short time to death, rapid assessment at ICU admission and immediate initiation of lifesaving interventions should be prioritized for all high-risk obstetric patients. Early identification of deterioration using structured monitoring tools can substantially reduce preventable deaths. These findings highlight the need for future prospective, multicenter studies that incorporate competing-risks analysis as a primary analytical approach when discharge alive is a relevant competing event for ICU mortality. Such studies should also evaluate targeted interventions addressing the identified key predictors and generate stronger evidence to inform clinical guidelines and national strategies aimed at reducing maternal mortality in low-resource settings.
